# lncRNA-disease association prediction based on matrix decomposition of elastic network and collaborative filtering

**DOI:** 10.1038/s41598-022-16594-5

**Published:** 2022-07-26

**Authors:** Bo Wang, RunJie Liu, XiaoDong Zheng, XiaoXin Du, ZhengFei Wang

**Affiliations:** College of Computer and Control, Qiqihar University, Qiqihar, 161006 China

**Keywords:** Bioinformatics, Computational biology and bioinformatics

## Abstract

In recent years, with the continuous development and innovation of high-throughput biotechnology, more and more evidence show that lncRNA plays an essential role in biological life activities and is related to the occurrence of various diseases. However, due to the high cost and time-consuming of traditional biological experiments, the number of associations between lncRNAs and diseases that rely on experiments to verify is minimal. Computer-aided study of lncRNA-disease association is an important method to study the development of the lncRNA-disease association. Using the existing data to establish a prediction model and predict the unknown lncRNA-disease association can make the biological experiment targeted and improve its accuracy of the biological experiment. Therefore, we need to find an accurate and efficient method to predict the relationship between lncRNA and diseases and help biologists complete the diagnosis and treatment of diseases. Most of the current lncRNA-disease association predictions do not consider the model instability caused by the actual data. Also, predictive models may produce data that overfit is not considered. This paper proposes a lncRNA-disease association prediction model (ENCFLDA) that combines an elastic network with matrix decomposition and collaborative filtering. This method uses the existing lncRNA-miRNA association data and miRNA-disease association data to predict the association between unknown lncRNA and disease, updates the matrix by matrix decomposition combined with the elastic network, and then obtains the final prediction matrix by collaborative filtering. This method uses the existing lncRNA-miRNA association data and miRNA-disease association data to predict the association of unknown lncRNAs with diseases. First, since the known lncRNA-disease association matrix is very sparse, the cosine similarity and KNN are used to update the lncRNA-disease association matrix. The matrix is then updated by matrix decomposition combined with an elastic net algorithm, to increase the stability of the overall prediction model and eliminate data overfitting. The final prediction matrix is then obtained through collaborative filtering based on lncRNA.Through simulation experiments, the results show that the AUC value of ENCFLDA can reach 0.9148 under the framework of LOOCV, which is higher than the prediction result of the latest model.

## Introduction

The human genome roughly contains more than 20,000 protein-coding genes, which account for about 2% of the human genome^1^. In addition, more than 98% of the genome cannot be compiled into proteins^1–3^, but tens of thousands of non-coding genes are also generated. Long non-coding RNA (lncRNA) is a type of non-coding RNA with a length greater than 200 nucleotides^4^ .lncRNA does not code for protein.Still, it plays a role in regulating gene expression at various levels of life activities, including genetic regulation, transcription regulation, cell differentiation, etc.^5^. In addition, the disorders and mutations of lncRNA are related to many complex human diseases, such as diabetes^6^, cardiovascular disease^7^, breast cancer^8^, and so on. Accumulating studies have shown that lncRNAs can regulate gene expression in many ways, and the variation in gene expression is important in complex diseases. Thus lncRNAs are associated with various human diseases. For example, lncRNA PCA3 is treated as a potential biomarker of prostate cancer^9^. lncRNA ‘BC200’expresses significantly higher in Alzheimer’s disease tissue compared to normal tissues^10^. The expression of lncRNA ‘BACE1-AS’ drives rapid feed-forward regulation of b-secretase in Alzheimer's disease^11^. lncRNA ‘H19’ not only has great effects on primary breast carcinomas^12,13^ but is also confirmed to be associated with lung cancer^14^. With the development of artificial intelligence technology and the maturity of big data technology, researchers can analyze and process known data to predict the potential relationship between lncRNA and diseases. Such methods can help people understand human diseases and contribute to the diagnosis and treatment of diseases^15^. In recent years, many methods have been adopted to predict the potential association between lncRNA and diseases, and good results have been achieved. According to different algorithm ideas, these methods can be divided into two categories: data integration methods based on biological networks and data integration methods based on machine learning models. Data fusion methods based on biological networks can be further divided into predicting lncRNA disease potential association based on lncRNA or disease attributes and predicting lncRNA-disease potential association based on multi-source data integration. Among them, in predicting the potential association between lncRNA and disease based on lncRNA or disease attributes, Chen et al.^16^developed the LRLSLDA computational model, which is a model for predicting potential disease-related lncRNAs based on a semi-supervised learning framework. The model is based on the assumption that similar diseases tend to be associated with lncRNAs with similar functions. LRLSLDA combines known disease-lncRNA associations and lncRNA expression profiles to obtain an AUC of 0.776 under leave-one-out cross-validation (LOOCV), while also requiring no information on negative samples, which are often difficult to obtain. But LRLSLDA still has some limitations. For example, there are many parameters in the model, and how to choose the parameters has not been fundamentally solved. Sun^17^ and others believe that lncRNA with similar functions will be associated with similar diseases. On this basis, a method based on a global network random walk (RWRlncd) is proposed to predict the association between lncRNA and disease. RWRlncd constructs a lncRNA functional similarity network and then uses the restart random walk method to predict the association between potential lncRNA and disease. However, this method only considers the lncRNA with known association with disease and does not consider the situation that there is no known association with any disease.Liu^18^ predicted the potential lncRNA-disease association by integrating the known human disease genes and gene lncRNA co-expression relationship. However, if there is no relevant gene association for a disease, the method can not predict the associated lncRNA.Zhou^19^ assumed that those lncRNA sharing significantly enriched interacting miRNA would be associated with similar diseases, and proposed a kind of RWRLDA method. RWRLDA integrates three types of networks: miRNA-related lncRNA-lncRNA association networks, disease similarity network, and lncRNA-disease association network into heterogeneous networks, and uses restart random walk to predict relevant disease information. In predicting the potential association between lncRNA and disease based on multi-source data integration, Chen^20^ proposed a prediction method based on multi-source data integration called KATZLDA. KATZLDA integrates the known lncRNA disease association information, lncRNA expression map, lncRNA functional similarity, disease semantic similarity, and Gaussian interaction kernel similarity matrix to predict lncRNA-disease association. Chen^21^ also proposed an improved restart random walk model (IRWRLDA) on lncRNA-disease association. IRWRLDA uses lncRNA-miRNA interaction information, miRNA-disease association, disease semantic similarity based on MESH terms, lncRNA expression map, and known lncRNA-disease association to predict unknown lncRNA disease association information. Lan^22^ proposes a method using graph attention networks(GANLDA) to extract useful information from tumor and disease features to predict lncRNA-disease potential associations. The above methods based on biological network and data integration do not consider the structural differences between the lncRNA network and disease network, but also ignore the important role of the special structure of the disease network in predicting lncRNA-disease association. Sheng^23^ addressed the above problems and proposed a model called VADLP to adaptively learn and integrate pairwise topology, node attributes, and deep feature distributions encoded from multi-source data to predict disease-related lncRNAs.In the data integration method based on a machine learning model, Wang^24^ proposed the asymmetric non-negative matrix cooperative decomposition method (S-NMTF) to realize the clustering of multi-type associated data sources. The data integration framework (DFMF) proposed by Zitnik^25^ uses the three-factor collaborative matrix decomposition technology to integrate various heterogeneous data sources. After decomposition and optimization, the low-rank representation of each biomolecule is obtained, and then the lncRNA and disease low-rank representation are used to reconstruct the lncRNA-disease association. Biswas^26^ developed the lncRNA-disease association prediction model (RIMC) based on matrix completion, which integrates a variety of heterogeneous and homogeneous data and uses the non-negative matrix decomposition method to predict the interaction between lncRNA and disease. The above methods based on matrix decomposition can maintain the internal structure of heterogeneous data sources. Liu^27^ established a new matrix factorization model to predict lncRNA-miRNA interactions, namely lncRNA-miRNA interaction prediction by logistic matrix factorization and neighborhood regularization (LMFNRLMI). The model utilizes only known positive samples to mine potential lncRNA-disease associations. Zeng^28^ proposed a hybrid computational framework (SDLDA) for lncRNA-disease association prediction. In this computational framework, Zeng uses singular value decomposition and deep learning to extract linear and nonlinear features of lncRNAs and diseases, respectively. The combination of linear and nonlinear features is mutually reinforcing, which is better than just using matrix factorization or deep learning. To overcome the limitations of matrix factorization, Lan^29^ developed a mixed model (named LDICDL) to predict the association between novel lncRNAs (or diseases) and diseases (or lncRNAs). However, due to the incompleteness of biological data and the limitations of model assumptions and experimental design, the existing lncRNA disease prediction methods still face many challenges. The above methods have their advantages and uniqueness. So far, many achievements have been made in the association prediction between lncRNA and disease. However, there are still some shortcomings. For example, the method based on biological network fusion depends on experimental data, and the amount of experimental data is too small, which will lead to the deviation of prediction results to a certain extent; The method based on machine learning lacks accurate negative samples, so there is an urgent need for reliable and effective methods to extract the most likely negative sample data. How to solve these problems and further improve the accuracy of model prediction is a challenge for future researchers. They did not take full advantage of known lncRNA signature data and disease signature data and did not consider the limitations of missing data and data overfitting on accuracy and predictive performance. This paper presents a novel computational framework (ENCFLDA) to predict the association of lncRNAs with the disease. It uses matrix factorization combined with an elastic net algorithm for prediction, which can make the prediction model more stable and eliminate the problem of data overfitting. Experimental results demonstrate that our method outperforms other state-of-the-art methods.

## Results

### Evaluation metrics

To evaluate the robustness and prediction performance of ENCFLDA, the AUC value calculated by Leaving One Cross Validation (LOOCV) is used as the evaluation index in this section. The model is compared with the current more advanced model, that is, CFNBC^30^, NBCLDA^31^, LMFP^32^, DMFLDA^33^. We take the relationship between each lncRNA and disease as the test set. By comparing the calculated results with the given threshold, we can also obtain a series of true positive rate (TPR) and false positive rate (FPR) according to the following formula :

1 $$TPR = \frac{TP}{{TP + FN}}$$

2 $$FPR = \frac{FP}{{FP + TN}}$$

**Figure 1:**
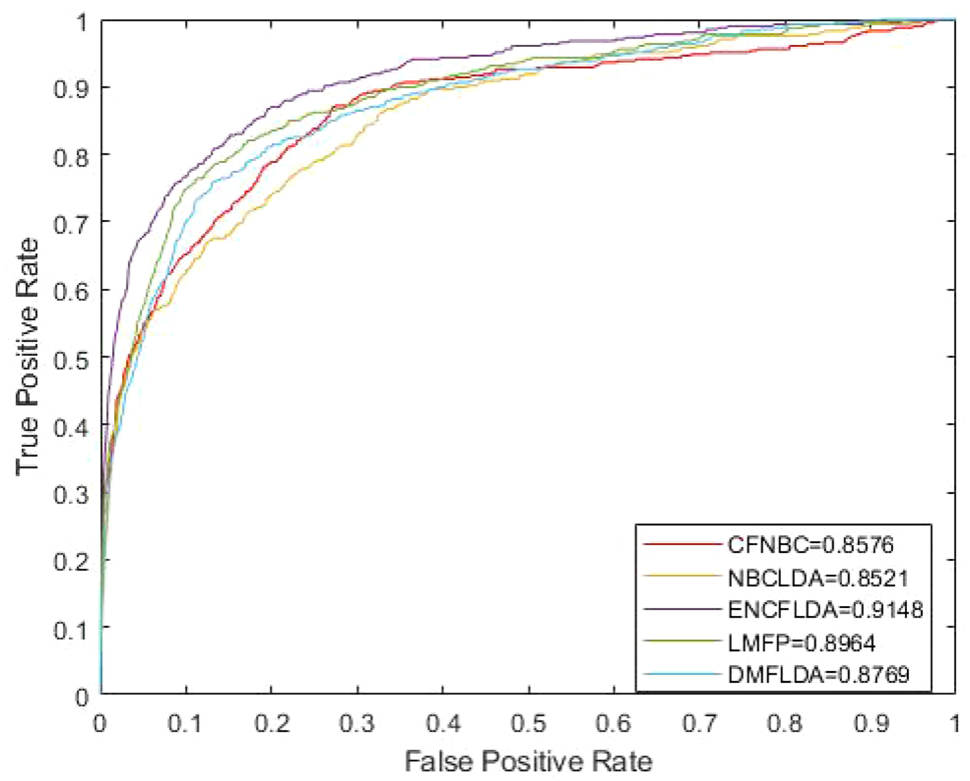
ROC comparison between ENCFLDA and other advanced models based on the same data set.

The true positive rate (TPR) and false positive rate (FPR) were used to draw the receiver operating characteristic curve (ROC), and the area under the ROC curve (AUC) was calculated to evaluate the model performance.AUC = 1 indicates that the model is perfect; 0.5 < AUC < 1 indicates that the model has predictive value; AUC = 0.5 indicates that the model is random model. Obviously, the closer the AUC value is to 1, indicating that the prediction ability of the model is accurate. The final results are shown in Fig. 1 below. It is easy to see that the model ENCFLDA proposed by us can reach the AUC value of 0.9148.

### Comparison with other methods

**Figure 2:**
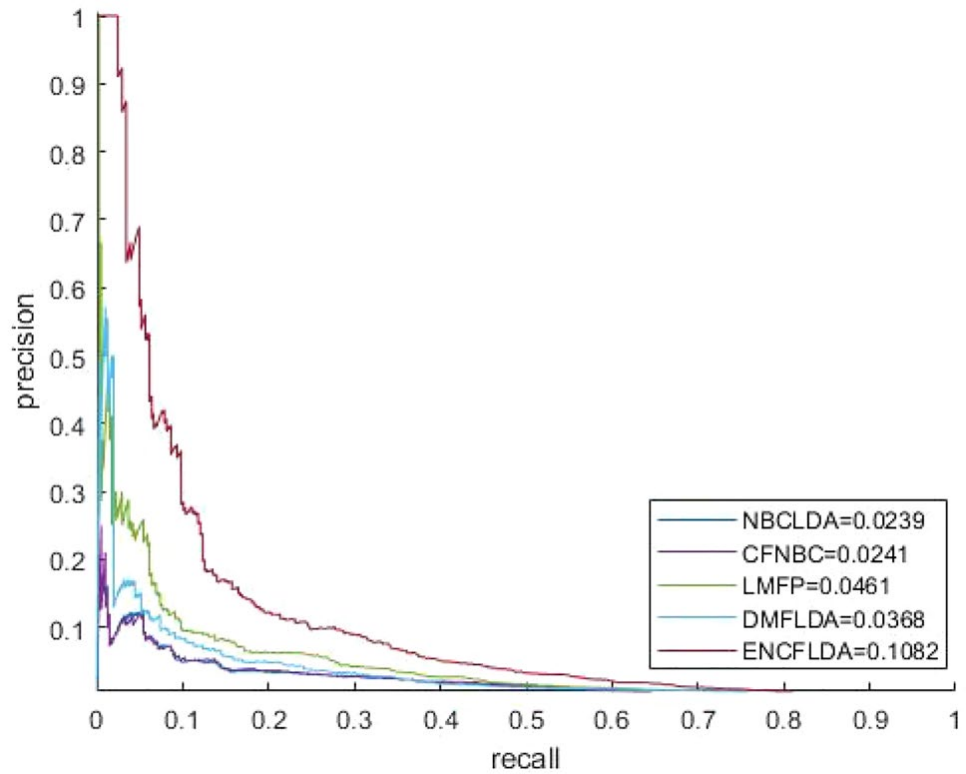
AUPR comparison between ENCFLDA model and other advanced models based on the same data set.

We compare ENCFLDA with four popular computational methods (CFNBC, NBCLDA, LMFP, and DMFLDA).We compare the five models based on the LOOCV framework, and the ROC comparison diagram is shown in Fig. 1. It is obvious that the AUC of ENCFLDA model is 0.9148, which is better than CFNBC(0.8576),NBCLDA(0.8521),LMFP(0.8964),DMFLDA(0.8769).The results show that the prediction effect of ENCFLDA model is better than other models. The AUPR comparison chart based on LOOCV is shown in Fig. 2.

### Analysis of parameters

**Figure 3:**
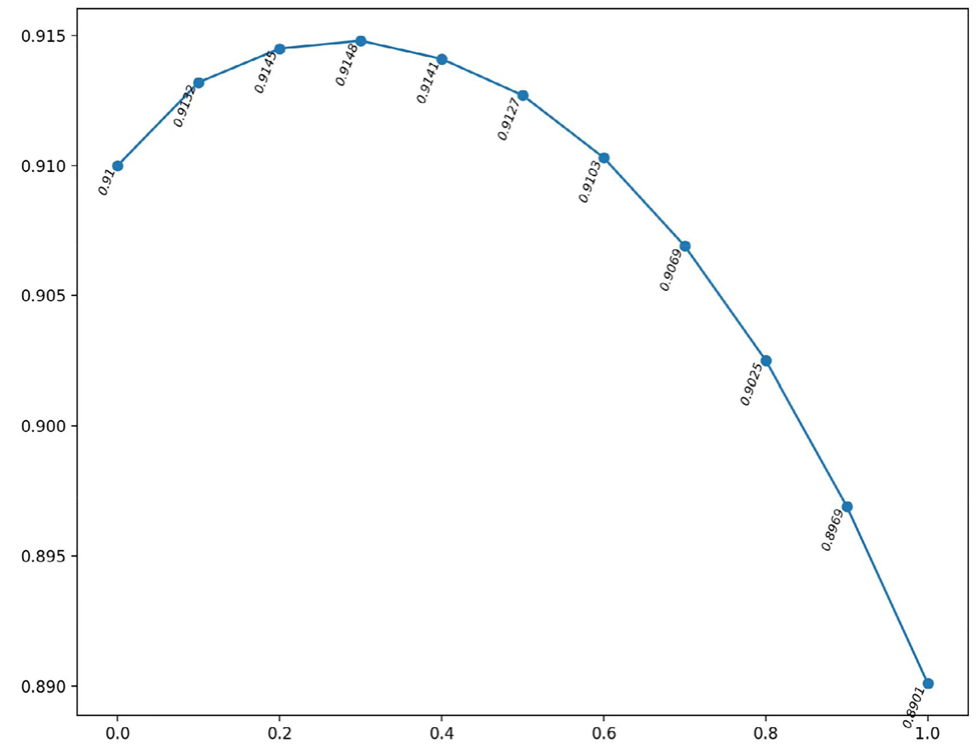
ROC under different parameters and Transformation curve of a parameter in the range of [0,1].

In this model, we introduce parameters . Its value range is [0,1]. This parameter is used to adjust the ratio in the elastic network calculation. We experimented with parameter 0 and incremented 0.1, and the results are shown in Fig. 3. It is not difficult to see that when = 0, AUC is 0.9100; When = 1, AUC is 0.8901; when = 0.3, AUC is 0.9148.The results are shown in Fig. 3.

### Ablation experiments

We conduct a set of ablation experiments to the contributions of cosine similarity-based KNN, matrix factorization incorporating elastic networks, and lncRNA-based collaborative filtering algorithms. The experimental results are shown in Table 1. Without KNN based on cosine similarity, the prediction performance of AUC and AUPR decreased by 3.05% and 7.39% compared to our final model. Without matrix factorization incorporating elastic nets, AUC and AUPR are 2.32% and 6.68% lower than our method. Compared with the model without lncRNA-based collaborative filtering, AUC and AUPR were 1.86% and 5.7% lower than our method.Ablation experiments demonstrate the critical and vital contributions of these three modules. The experimental results show that the contribution of KNN based on cosine similarity is the most significant among the three modules. One of the possible reasons is that the datasets used in the lncRNA-disease association prediction process have the characteristics of single and few features. As the input of lncRNA-disease association prediction will lead to inaccurate prediction results or fall into the optimum local problem. The KNN algorithm based on cosine similarity completes the missing data. The contribution of matrix factorization incorporating elastic nets is the second largest. The model solves the problem of biased prediction caused by the inherent logical relationship between lncRNAs and diseases. The elastic network algorithm is added to the matrix decomposition, which effectively improves the prediction of the relationship between unknown lncRNAs and diseases by matrix decomposition, and improves the stability of the model.

**Table 1:** The contributions of all components of the proposed method.

KNN based on cosine similarity	Matrix decomposition	Collaborative filtering	AUC	AUPR
×	√	√	0.8843	0.0343
√	×	√	0.8916	0.0414
√	√	×	0.8962	0.0512
√	√	√	0.9148	0.1082

### Case studies

**Figure 4:**
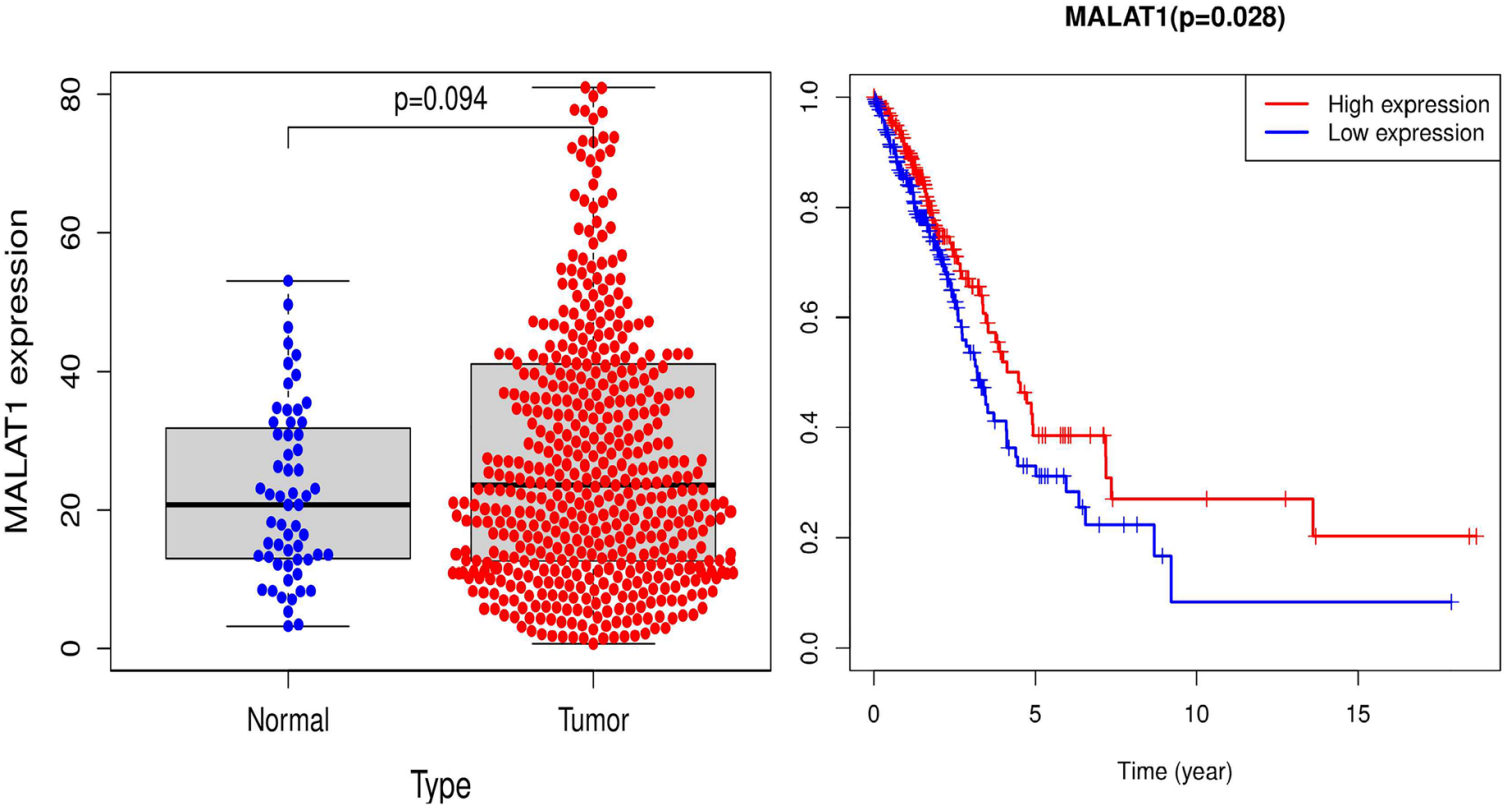
Differentiated expression and Survival period of genes in the normal and tumor sample.

**Figure 5:**
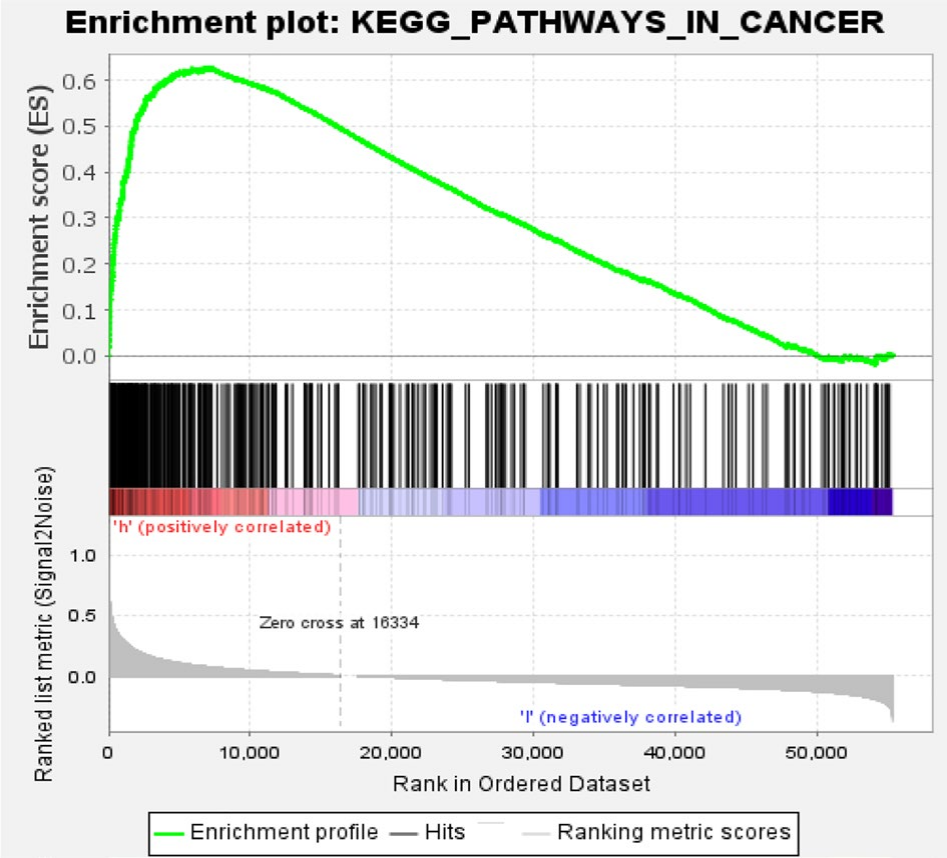
Enriched gene sets in small cell lung cancer, the KEGG gene sets, by samples of high gene expression.

In this section, we conducted a case study based on the above experiments to further verify the prediction performance of ENCFLDA. During the simulation, for each given disease, the potentially relevant lncRNA predicted by ENCFLDA will be classified according to their expected values, and the scores are arranged in descending order. In this section, we selected two cases of breast cancer and lung cancer as treatment targets. It is verified by references, as shown in Table 2. In recent years, lung cancer has been the leading cause of cancer death worldwide. Histopathologically, lung cancer is mainly divided into non-small cell lung cancer (NSCLC) and small cell lung cancer (SCLC)^34^. Recent studies suggest that lncRNAs play an essential role in the occurrence and development of lung cancer^35^. Therefore, we will take lung cancer as an example and use the ENCFLDA computational model to predict potential lung cancer-related lncRNAs.The results are shown in Table 1. It can be seen that 9 of the top 15 potential lung cancer-related lncRNAs predicted by our model have been confirmed by authoritative biological experiments. Among them, MALAT1 is highly correlated with lung cancer metastasis^36,37^, which will promote the movement of lung cancer cells by regulating the expression of movement-related genes^38^. It can be an essential biomarker for the development of lung cancer metastasis^39^.OIP5-AS1 is strongly expressed in lung cancer tissues and is related to tumor size and tumor growth rate^40^. As for breast cancer, according to the relevant literature, it is very common in women^41,42^. Studies have shown that lncRNAs play an important role in the occurrence and development of breast cancer^43,44^. Therefore, predicting related lncRNAs as breast cancer risk genes, diagnostic markers, and prognostic markers is very important for the treatment and diagnosis of breast cancer. The downregulation of H19 will significantly reduce colony formation and non-anchored growth of breast cancer and lung cancer cells. Next, we took the MALAT1 gene as an example for further analysis to verify whether it might be associated with lung cancer. In our study, we divided all lung cancer patient samples into high and low expression groups. This phenomenon was observed by survival analysis. That, the survival time of lung cancer patients in the MALAT1 gene high expression group was relatively short, as shown in Fig. 4. Furthermore, further results showed that the expression of these genes in cancer samples was significantly higher than that in normal samples, as shown in Fig. 4. Based on the above results, we finally concluded that the expression of these genes was significantly positively correlated with the survival time and clinicopathological characteristics of lung cancer patients. In addition, GSEA enrichment analysis also showed that the group with high MALAT1 gene expression was mainly enriched in the process of small cell lung cancer, as shown in Fig. 5.

**Table 2:** Candidate lncRNAs and TWO rank in the top 15 of the TWO cases and the related literature.

Disease	lncRNA	Evidence(PMID)	Rank
Lung Neoplasms	XIST	29130102,31632059	1
Lung Neoplasms	MALAT1	23243023	3
Lung Neoplasms	KCNQ1OT1	30471108	4
Lung Neoplasms	OIP5-AS1	32774481	6
Lung Neoplasms	NEAT1	28615056	7
Lung Neoplasms	HCG18	32559619	8
Lung Neoplasms	DCP1A	32034313	9
Lung Neoplasms	SNHG16	31071307	11
Lung Neoplasms	FGD5-AS1	31919528	13
Breast Neoplasms	OIP5-AS1	32945479	3
Breast Neoplasms	SNHG16	32945479	5
Breast Neoplasms	SCAMP1	29497041	6
Breast Neoplasms	FGD5-AS1	33880593	13
Breast Neoplasms	LINC00657	32996041	14
Breast Neoplasms	TUG1	28950664	15

## Discussions

In recent years, with the deepening of research, more and more pieces of evidence have shown that lncRNAs play an essential role in tumor proliferation, apoptosis, invasion, and prognosis. It requires a lot of human resources and material resources. Therefore, integrating the potential data associations of biology and using existing algorithms to develop accurate and efficient computational models to predict potential lncRNA-disease associations is the development trend of such research. To predict potential lncRNA-disease associations, we propose a novel computational model, termed ENCFLDA. The first step in the model was to integrate existing miRNA-disease associations, lncRNA-disease associations, and lncRNA-miRNA associations into a new lncRNA-disease association matrix. Then, based on the newly constructed association matrix, the lncRNA-disease association matrix was obtained and the weighted network was updated through cosine similarity, and the KNN algorithm. Finally, we can use our obtained association matrix to build our model ENCFLDA to predict potential associations between lncRNAs and diseases. In addition, case studies of breast and lung cancer have also demonstrated that ENCFLDA models have high accuracy in predicting underlying lncRNA disease associations. In recent years, many lncRNA-disease prediction models have emerged. Most of these models directly exploit the association information between lncRNAs and diseases to predict unknown lncRNA-disease associations. But this approach has drawbacks. When we now use lncRNA-disease associations, the matrix is too sparse, resulting in a lack of confidence in the computational results and model instability. Therefore, we had to add miRNA nodes to re-establish some significant associations that were not present in the lncRNA disease dataset and to incorporate elastic network algorithms. This way, the problem of missing lncRNA-disease association information can be addressed.

## Conclusion

In this paper, we introduce a matrix decomposition combined with an elastic network and collaborative filtering method (ENCFLDA) to predict the association between lncRNA and disease. The model has a good effect on sparse models with few weights. It can not only delete invalid features but also has good stability. Compared with other methods, ENCFLDA performs better in AUC in the loocv scheme. Other important reference indicators also show the perfect performance of ENCFLDA. To further verify the accuracy of ENCFLDA, we predicted two kinds of diseases (lung cancer and breast cancer) according to the prediction results of ENCFLDA. Taking the MALAT1 as an example, GSEA enrichment analysis, difference analysis, and other means are used to verify the accuracy of the prediction model. The excellent performance of the ENCFLDA method is mainly due to the following reasons. Firstly, the ENCFLDA model has a good effect on sparse models with few weights. It can not only delete invalid features but also has good stability. Secondly, the single similarity between lncRNA and disease is calculated, which provides us with rich biological information. Finally, through the optimization model of collaborative filtering, the final lncRNA-disease related prediction matrix is obtained, and the prediction results of the matrix are well optimized.

## Methods

### Dataset preprocessing

**Figure 6:**
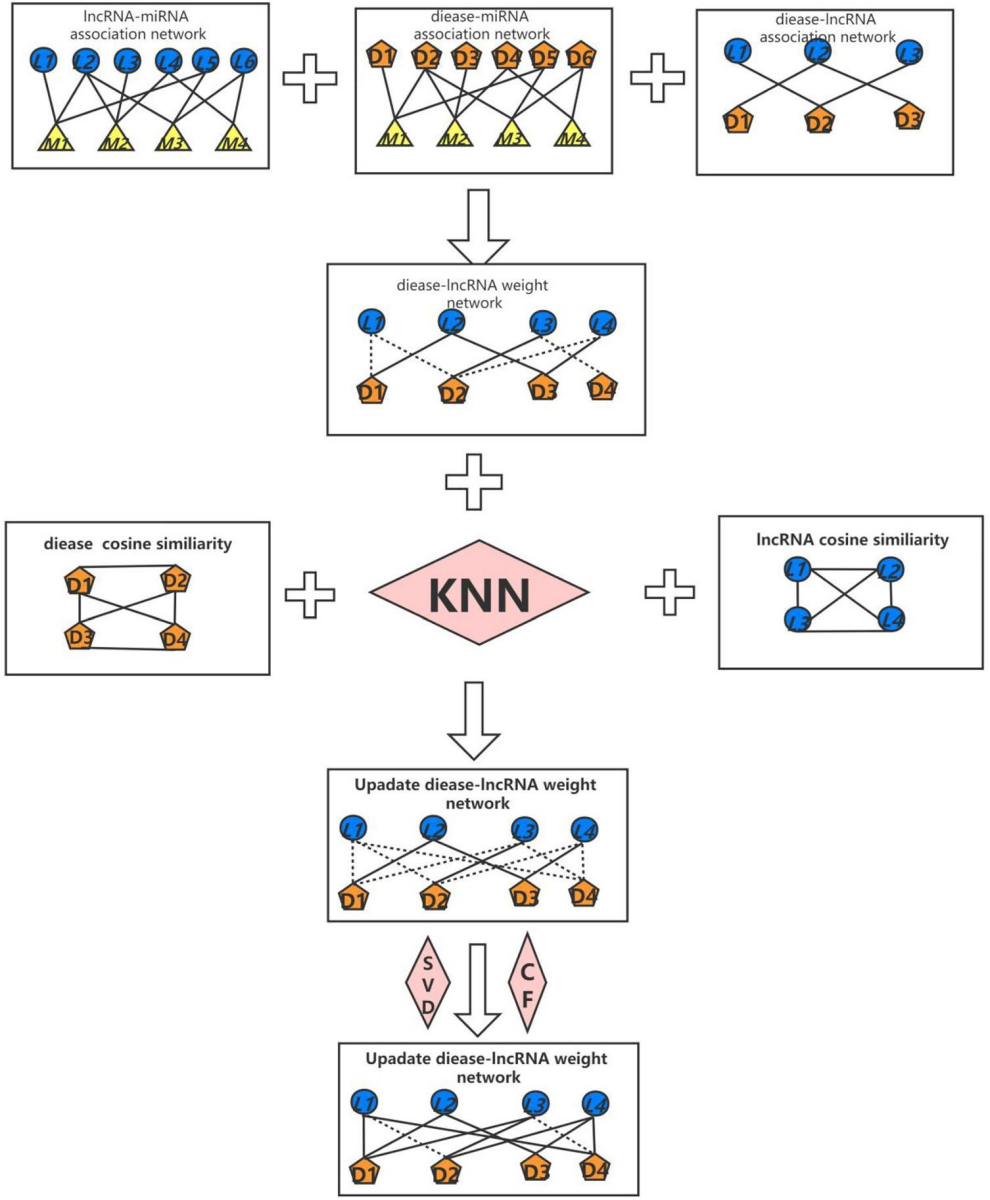
Flow Chart of ENCFLDA Applied to lncRNA-Disease Association Prediction.

First, we downloaded the known lncRNA-disease association datasets from MNDRv2.0 database^45^ (2017 Edition),which contains 1089 lncRNAs and 373 diseases.The available information includes 4073 miRNA–disease associations extracted from HMDD database ^46^(2018 Edition) and 9086 lncRNA–miRNA interactions obtained from Starbase v2.0 database^47^ (2015 Edition). Second, we downloaded lung cancer gene transcriptome data and clinical data through the TCGA database. The above datasets are all from authoritative public databases. The obtained data were preprocessed, and finally the miRNA-disease adjacency matrix $A_{MD}$ and the lncRNA-miRNA adjacency matrix $A_{LM}$ were constructed. Among them, when the two data have a known relationship, we assign a value of 1, and when the two data have no known relationship, we assign a value of 0. The experimental steps are shown in Fig. 6.

### Construct adjacency matrix of lncRNA-disease association matrix

Using the processed lncRNA-miRNA adjacency matrix $A_{LM}$ and miRNA-disease association adjacency matrix $A_{MD}$ to calculate the lncRNA-disease association matrix, the method is as follows:

3 $$A_{LD} = A_{LM} *A_{MD}$$

### Cosine similarity for diseases

The cosine similarity for diseases between lncRNA-diseases adjacency matrix was calculated:

4 $$CD\left( {i,j} \right) = \frac{{A_{LD} \left( {:,i} \right)*A_{LD} \left( {:,j} \right)}}{{\left\| {A_{LD} \left( {:,i} \right)} \right\|\left\| {A_{LD} \left( {:,j} \right)} \right\|}}$$

### Cosine similarity for lncRNA

The cosine similarity for lncRNA between lncRNA-diseases adjacency matrix was calculated:

5 $$CL\left( {i,j} \right) = \frac{{A_{LD} \left( {i,:} \right)*A_{LD} \left( {j,:} \right)}}{{\left\| {A_{LD} \left( {j,:} \right)} \right\|\left\| {A_{LD} \left( {j,:} \right)} \right\|}}$$

### Calculation of KNN algorithm based on cosine similarity

Considering that the known lncRNA disease association is very sparse, this may lead to the existence of some lncRNAs unrelated to any disease, or some diseases unrelated to any lncRNA.Consequently, some potential associations between predicted lncRNA and disease will be ineffective.Therefore, we will use the weighted KNN to make the matrix less sparse. First, the i-th row of matrix $A_{LD}$ is expressed as $A_{LD} \left( {l_{i} ,:} \right)$ and the j-th column of matrix $A_{LD}$ is expressed as $A_{LD} \left( {:,d_{j} } \right)$. According to the above formula (3), we can obtain the cosine similarity of lncRNA, so that we can update the formula:

6 $$A_{LD} \left( {:,d} \right) = \sum\limits_{i \in [1,K]} {CD\left( {i,j} \right)*A_{LD} } \left( {:,d_{j} } \right)$$

According to the above formula (4), we can obtain the cosine similarity of the disease,and then, we can update the formula:

7 $$A_{LD} \left( {l,:} \right) = \sum\limits_{i \in [1,K]} {CL\left( {i,j} \right)*A_{LD} \left( {l_{i} ,:} \right)}$$

### Establishment of ENCFLDA prediction model

So far, matrix decomposition technology has been widely used in the field of recommendation systems. It can not only reduce the computational complexity through matrix decomposition, but also have good performance in solving the problem of matrix scarcity. The purpose of matrix decomposition combined with elastic network is to find two low-level potential characteristic matrices, and their products are used to fit the original matrix. Therefore, for the weight matrix $A_{LD} \in R^{{{\text{n}}_{{\text{l}}} \times {\text{n}}_{{\text{d}}} }}$ constructed above, it is obvious that we can decompose $A_{LD}$ into two different matrices $U \in R^{{{\text{n}}_{{\text{l}}} \times {\text{k}}}}$ and $V \in R^{{{\text{n}}_{{\text{d}}} \times {\text{k}}}}$. After that, the disease-related lncRNA prediction problem can be further expressed by the following formulas (8) and (9):

8 $${\text{arg}}\mathop {{\text{min}}}\limits_{U,V} \sum\limits_{{{\text{i}} = {1}}}^{{{\text{n}}_{{\text{l}}} }} {\sum\limits_{{{\text{j}} = {1}}}^{{{\text{n}}_{{\text{d}}} }} {\left( {A_{LD} \left( {{\text{i}},j} \right) - \mathop {A_{LD} \left( {i,j} \right)}\limits^{ \wedge } } \right)^{2} } }$$

9 $$\mathop {A_{LD} }\limits^{ \wedge } \left( {i,j} \right) = \sum\limits_{k} {U_{ik} *V_{jk} = \sum\limits_{k} {U_{ik} *V_{kj}^{T} = U_{i} V_{j}^{T} } }$$

**Figure 7:**
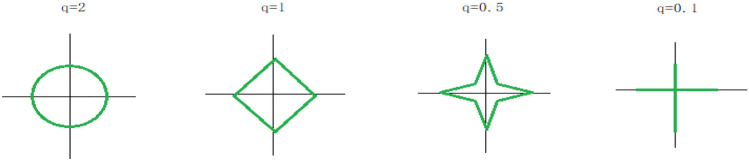
Constraint domain of ridge regression.

Elastic network is a linear regression model trained with L1 and L2 norms as a priori regular terms. Elastic network is beneficial when many features are interrelated. Lasso is likely to consider only one of these features randomly, while elastic networks prefer to choose two.In practice, one advantage of the trade-off between lasso and ridge is that it allows the stability of ridge to be inherited during the cycle. The elastic network contains two parameters, namely mixed parameter ratio $\alpha$ and penalty parameter $\lambda$. The elastic network adjusts the convex combination of L1 and L2 through mixed parameter ratio $\alpha$, and selects the variables with the value of penalty parameter $\lambda$, so as to select the variables and maintain the stability of the model.The penalty function can be expressed as: $\lambda \sum\limits_{{}}^{p} {\left| {w_{t} } \right|^{q} }$. When $q$ has different values, it represents different penalty terms, and $q = 1$ represents L1 norm, that is, the constraint domain of lasso regression;$q = {2}$ represents L2 norm, that is, the constraint domain of ridge regression. It can be seen from the figure below that when the values of $q$ are different, the range of constraint domain and the strength of constraint are also different. The scope of its constraint domain can be observed through Fig. 7.Obviously, the above formula (8) and formula (9) constitute a convex optimization problem, which can be easily solved by some existing optimization algorithms such as gradient descent method.After we join the elastic network, the loss function will be updated and expressed by formula (10).For convenience, we let $\mathop {A_{LD} }\limits^{{}} \left( {i,j} \right) = \psi_{ij}$:

10 $$L\left( {U,V} \right) = \sum\limits_{{\left( {i,j} \right) \in k}} {(\psi_{ij} - U_{i} V_{j}^{T} )}^{2} + \lambda_{1} \left\| {U_{i} } \right\| + \lambda_{2} \left\| {U_{i} } \right\|^{2} + \lambda_{1} \left\| {V_{j} } \right\| + \lambda_{2} \left\| {V_{j} } \right\|^{2}$$

Let $\lambda = \lambda_{{1}} + \lambda_{{2}}$ and $\alpha = \frac{\lambda }{{\lambda_{{1}} + \lambda_{{2}} }}$ According to the above description, we can get the following formula (11):

11 $$L\left( {U,V} \right) = \sum\limits_{{\left( {i,j} \right) \in k}} {(\psi_{ij} - U_{i} V_{j}^{T} )}^{2} + \lambda \sum\limits_{i}^{{}} {(\alpha \left| {U_{i} } \right| + (1 - \alpha )U_{i}^{2} } ) + \lambda \sum\limits_{j}^{{}} {(\alpha \left| {V_{j} } \right| + (1 - \alpha )V_{j}^{2} } )$$

From the formula (9), the penalty function of elastic network is:

12 $$\lambda \sum\limits_{{}}^{{}} {(\alpha \left| {w_{t} } \right| + (1 - \alpha )w_{t}^{2} } )$$

The value range of mixed parameter ratio $\alpha$ of elastic network is 0 to 1. When $\alpha$ is 0, the elastic network regression becomes ridge regression, and when $\alpha$ is 1, the elastic network becomes lasso regression. In this experiment = 0.3.According to the properties of elastic network, the formula is rewritten into Lagrange function form, which can be rewritten into the following form (13):

13 $$\frac{\partial L}{{\partial U_{i} }} = \sum\limits_{j} {2\left( {U_{i}^{T} V_{j} - \psi_{ij} } \right)V_{j} + \sum\limits_{i} {\lambda \left( {\alpha + 2\left( {1 - \alpha } \right)U_{i} } \right)} }$$

Then, according to the random gradient descent method, the parameters need to advance along the fastest descent direction. Therefore, the following recurrence formula (14) can be obtained:

14 $$U_{i} = U_{i} - \sum\limits_{j} {2\left( {U_{i}^{T} V_{j} - \psi_{ij} } \right)V_{j} + \sum\limits_{i} {\lambda \left( {\alpha + 2\left( {1 - \alpha } \right)U_{i} } \right)} }$$

Similarly, we can get:

15 $$V_{j} = V_{j} - \sum\limits_{i} {2\left( {U_{i}^{T} V_{j} - \psi_{ij} } \right)U_{i} + \sum\limits_{j} {\lambda \left( {\alpha + 2\left( {1 - \alpha } \right)V_{j} } \right)} }$$

Finally, we use the lncRNA-based collaborative filtering algorithm to calculate the score matrix, and the score between the lncRNA-disease predicted by ENCFLDA will depend on the common neighbors between the lncRNA and the disease. After previous processing, the association between lncRNA-disease is not sparse. Therefore, the similarity matrix $Sim\left( {i,j} \right)$ can be calculated as follows:

16 $$Sim(i,j) = \frac{i \cdot j}{{\left\| i \right\| \cdot \left\| j \right\|}}$$

Then, the obtained similarity matrix can be used to calculate the final score matrix of ENCFLDA, and the formula is as follows:

17 $$ENCFLDA\left( {i,j} \right) = \frac{{\sum {\left( {Sim(i,j) \cdot \psi \left( {i,j} \right)} \right)} }}{{\sum {Sim(i,j)} }}$$

$ENCFLDA\left( {i,j} \right)$ is the final association score between lncRNA i and disease j.

## Data availability

The datasets generated during the current study are available in the HMDDrepository, http://www.cuilab.cn/; starBaserepository, https://starbase.sysu.edu.cn/index.php; TCGA repository, https://portal.gdc.cancer.gov/; GitHub: https://github.com/arejay1998/ENCFLDA.
